# *P*-Wave Area Predicts New Onset Atrial Fibrillation in Mitral Stenosis: A Machine Learning Approach

**DOI:** 10.3389/fbioe.2020.00479

**Published:** 2020-05-15

**Authors:** Gary Tse, Ishan Lakhani, Jiandong Zhou, Ka Hou Christien Li, Sharen Lee, Yingzhi Liu, Keith Sai Kit Leung, Tong Liu, Adrian Baranchuk, Qingpeng Zhang

**Affiliations:** ^1^Tianjin Key Laboratory of Ionic-Molecular Function of Cardiovascular Disease, Department of Cardiology, Tianjin Institute of Cardiology, Second Hospital of Tianjin Medical University, Tianjin, China; ^2^Xiamen Cardiovascular Hospital, Xiamen University, Xiamen, China; ^3^Laboratory of Cardiovascular Physiology, Li Ka Shing Institute of Health Sciences, Shatin, China; ^4^School of Data Science, City University of Hong Kong, Kowloon, China; ^5^Faculty of Medicine, Newcastle University, Newcastle, United Kingdom; ^6^Aston Medical School, Aston University, Birmingham, United Kingdom; ^7^Heart Rhythm Service, Kingston General Hospital, Queen's University, Kingston, ON, Canada

**Keywords:** mitral stenosis, mitral valve, *P*-wave area, decision tree, machine learning

## Abstract

**Introduction:** Mitral stenosis is associated with an atrial cardiomyopathic process, leading to abnormal atrial electrophysiology, manifesting as prolonged *P*-wave duration (PWD), larger *P*-wave area, increased *P*-wave dispersion (PWD_max_—PWD_min_), and/or higher *P*-wave terminal force on lead V1 (PTFV1) on the electrocardiogram.

**Methods:** This was a single-center retrospective study of Chinese patients, diagnosed with mitral stenosis in sinus rhythm at baseline, between November 2009 and October 2016. Automated ECG measurements from raw data were determined. The primary outcome was incident atrial fibrillation (AF).

**Results:** A total 59 mitral stenosis patients were included (age 59 [54–65] years, 13 (22%) males). New onset AF was observed in 27 patients. Age (odds ratio [OR]: 1.08 [1.01–1.16], *P* = 0.017), systolic blood pressure (OR: 1.03 [1.00–1.07]; *P* = 0.046), mean *P*-wave area in V3 (odds ratio: 3.97 [1.32–11.96], *P* = 0.014) were significant predictors of incident AF. On multivariate analysis, age (OR: 1.08 [1.00–1.16], *P* = 0.037) and *P*-wave area in V3 (OR: 3.64 [1.10–12.00], *P* = 0.034) remained significant predictors of AF. Receiver-operating characteristic (ROC) analysis showed that the optimum cut-off for *P*-wave area in V3 was 1.45 Ashman units (area under the curve: 0.65) for classification of new onset AF. A decision tree learning model with individual and non-linear interaction variables with age achieved the best performance for outcome prediction (accuracy = 0.84, precision = 0.84, recall = 0.83, *F*-measure = 0.84).

**Conclusion:** Atrial electrophysiological alterations in mitral stenosis can detected on the electrocardiogram. Age, systolic blood pressure, and *P*-wave area in V3 predicted new onset AF. A decision tree learning model significantly improved outcome prediction.

## Introduction

Inter-atrial block (IAB) results from impaired conduction of action potentials along Bachmann's bundle that connects the right and left atria (Tse et al., 2016). It is characterized electrocardiographically by a prolonged *P*-wave duration of >120 ms. This condition results in delayed and asynchronous activation of the left atrium (Agarwal et al., 2003; Budeus et al., 2005; Caldwell et al., 2014). IAB has been associated with higher incidence of stroke as well as cardiovascular and all-cause mortality (Ariyarajah et al., 2007; Magnani et al., 2011). However, it is unclear any benefit derived from early initiation of anti-coagulation in IAB before the development of atrial fibrillation (AF), and the risk may differ depending on the severity of IAB and the presence of other cardio-metabolic co-morbidities. Two other measures have been used to assess atrial electrophysiological remodeling. Firstly, *P*-wave dispersion, defined as the difference between maximum and minimum *P*-wave duration (PWD), is a measure of heterogeneous and discontinuous atrial activation. Secondly, *P*-wave terminal force in V1 (PTFV1) is a marker of left atrial disease independently of structural or pressure changes in the left atrium (Morris and Thompson, 1964) and has been shown to be a predictor of future incident AF (Martin Garcia et al., 2012). Prolonged PWDs, measured from amplified and digitized ECG signals obtained in sinus rhythm, predicted AF recurrence after pulmonary isolation procedures (Jadidi et al., 2018). Moreover, the area of the *P*-wave initial portion was independently associated with the development of AF in patients with left atrial overload (Ishida et al., 2010). AF complexity parameters derived from the ECG also predicted long-term outcomes following catheter ablation (Lankveld et al., 2016).

Mitral stenosis is a valvular disease frequently seen in parts of Asia, causing significant morbidity and mortality. In this condition, the most common arrhythmia encountered is AF, but there are limited data on electrocardiographic changes that reflect ongoing atrial cardiomyopathic process that precedes the development of fibrillation. Mitral stenosis patients have longer PWDs and higher *P*-wave dispersion than control subjects without mitral stenosis (Guntekin et al., 2008). Another study confirmed this observation and further demonstrated a significant correlation between maximum PWDs and left atrial size, transmitral valve gradient, and a negative correlation with mitral valve area (Rezaian et al., 2007). PTFV1 is higher in mitral stenosis and is a predictor of disease severity (Yuce et al., 2011). However, there are limited published data regarding the incidence of IAB, the relative contributions of partial and advanced IAB, and whether these indices predict incident AF in mitral stenosis.

## Methods

This study received approval from The Joint Chinese University of Hong Kong—New Territories East Cluster Clinical Research Ethics Committee. Clinical and electrocardiographic details of a cohort of Chinese patients referred to our center, which is a tertiary referral center and teaching hospital, between November 2009 and October 2016, for echocardiography, were analyzed retrospectively. Inclusion criteria were mitral stenosis patients with raw ECG data files available for analysis.

### Definitions, Data Extraction, Electrocardiographic Measurements, and Primary Outcome

**Figure 1:**
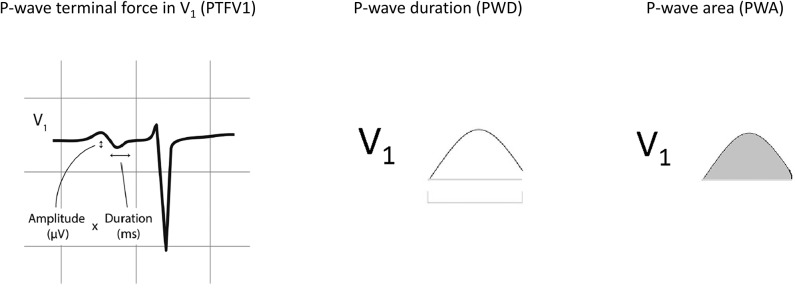
Normal inter-atrial conduction, partial and advanced inter-atrial block and left atrial enlargement. *P*-wave terminal force in V1 (PTFV1), *P*-wave duration (PWD), and *P*-wave area. Adapted from (He et al., 2017) with permission.

The following clinical details were obtained from the patients: age, gender, blood pressure, smoking status, diabetes mellitus, hypertension, hypercholesterolemia, and ischemic heart disease. For electrocardiographic parameters, data were extracted from patients who had ECGs that did not show atrial fibrillation (AF). The following parameters were manually measured by two investigators from the ECGs showing sinus rhythm. The following *P*-wave variables were determined from ECGs of patients in sinus rhythm. Automated measurements from raw ECG data were extracted from the Philips ECGVue program (Standard Edition). The ECG waveform data is captured at a sample rate of 4 MHz and reduced to 500 samples per second with 5 μV resolution. The mean, minimum, maximum, and standard deviation of different *P*-wave variables were calculated from values from all 12 leads (Figure 1). *P*-wave dispersion was defined as the maximum difference in PWD. *P*-wave terminal force in V1 (PTFV1) was defined as the area subtended by the terminal negative component of a biphasic *P*-wave in lead V1, with the area calculated by multiplication of the duration and depth of the waveform (He et al., 2017). The primary endpoint of this study was new onset persistent or permanent atrial fibrillation (AF). Paroxysmal AF at baseline or detected follow-up was excluded. The endpoint was met if AF was detected in at least two ECGs on follow-up 1 year apart in an absence of sinus rhythm detection in the intervening period.

### Statistical Analysis, Non-linear Variables, and Decision Tree Learning

Data were expressed as median [lower quartile to upper quartile]. Categorical data were analyzed by Fisher's exact test. Differences between study groups were tested using Kruskal-Wallis ANOVA. *P* < 0.05 was considered statistically significant. Non-linear interactions (e.g., interactions formed by some important individual variable) play an important role in predicting the outcome. The consideration of non-linear interactions overcomes linear model's assumption that the dependent and independent variables are linearly related. In this study, the logarithmic form of the multiplication non-linear items formed by age (important individual variable) and other continuous variables were considered, i.e., log(age${}^{*}x_{i}$), where *x*_*i*_ denotes the *i*th continuous variable. The adoption of logarithmic transformation is to obtain equivalent inference on variable-outcome associations while avoid the bias due to exponentiation on some squared and cubed variables. The non-linear variables considered were log(age^*^systolic blood pressure), log(age^*^diastolic blood pressure), log(age^*^diabetes mellitus), log(age^*^hypercholesterolaemia, log(age^*^ischaemic heart disease), log(age^*^left atrial diameter), log(age^*^mitral valve area), log(age^*^mitral valve gradient), log(age^*^mitral stenosis severity), log(age^*^ *P*-wave area in v3). For instance, for a patient whose age is 58 years old and has systolic blood pressure 110 mmHg, we generate the value of non-linear variable log(age^*^systolic blood pressure) by calculating log(58^*^110) = 3.8048. The values for the other non-linear variables are obtained in a similar way. Then these non-linear variables and the individual variables are together used as input in the risk prediction model. The reproduction of the non-linear variables can be obtained since there exists one-one mapping between the non-linear variables and the individual variable pairs that form them.

**Figure 2:**
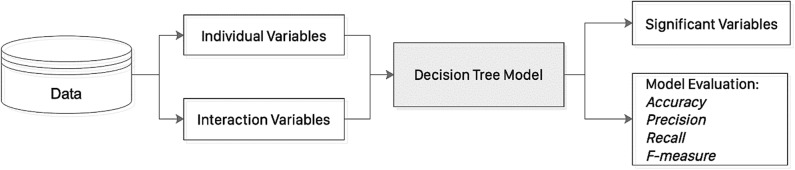
An illustration of the decision tree learning approach.

Decision tree learning uses a decision tree module (as a predictive model) to determine the outcome (or target value, represented by leaves) of a sample based on the associated observations (represented by branches) for model classification and prediction. The principles of decision tree are illustrated in Figure 2. In this study, a decision tree learning approach (classification and regression tree, CART Rutkowski et al., 2014 was used to predict new onset AF. Specifically, the non-linear variables (including log(age^*^systolic blood pressure), log(age^*^diastolic blood pressure), log(age^*^diabetes mellitus), log(age^*^hypercholesterolaemia), log(age^*^ischaemic heart disease), log(age^*^left atrial diameter), log(age^*^mitral valve area), log(age^*^mitral valve gradient), log(age^*^mitral stenosis severity), log(age^*^ *P*-wave area in v3) together with individual variables (including sex, age, systolic blood pressure, diastolic blood pressure, diabetes mellitus, hypercholesterolaemia, ischaemic heart disease, left atrial diameter, mitral valve area, mitral valve gradient, mitral stenosis severity, *P*-wave area in v3) were used as input to the DTL model, in order to predict the new onset of AF outcome in mitral stenosis. In the DTL model, leaves represent class label of new onset AF and branches represent feature conjunctions (both of non-linear variables and individual variables) that lead to new onset AF. DTL uses Gini index to construct a decision tree, which is calculated by the formula $Gini=1-\overset{C}{\underset{i=1}{\sum }}{\left(p_{i}\right)}^{2}$ representing the probability of a particular patient being wrongly classified when it is randomly chosen and *c* denotes the number of class (*c* = 2 for new onset AF classification in this study). The Gini index is used to create split points by considering a binary split for each variable in DTL. It varies between 0 and 1, where 0 denotes that all elements belong to a certain class or if there exists only one class, and 1 denotes that the elements are randomly distributed across various classes. A Gini Index of 0.5 denotes equally distributed elements into some classes.

Here we present an example to show the construction process of DTL with Gini index. Firstly, the frequency table of new onset AF outcome (27 with “yes” and 32 with “no”) was considered: there are 19 of 46 females with new onset AF, while eight of 13 males with new onset AF. The Gini index for sex as a decision node to split the tree was calculated: Gini index (new onset AF, male) = 2^*^(8/13)^*^(1–8/13) =0.4734, Gini index (new onset AF, female) = 2^*^(19/46)^*^(1–19/46) = 0.4849. In addition, the frequency table of ischaemic heart disease was considered: eight of 12 with ischaemic heart disease had new onset AF, and 19 of 47 without ischaemic heart disease had new onset AF. The corresponding Gini index was calculated by Gini(new onset AF, ischaemic heart disease as “yes”) = 2^*^(8/12)^*^(1–8/12)=0.4444, Gini index(new onset AF, ischaemic heart disease as “no”)=2^*^(19/47)^*^(1–19/47) = 0.4817. In the same manner, the Gini indices of all the variables (the Information Gain of a continuous variable, such as age, was discretized first) was calculated. DTL splits the dataset on different variables by referring to the Gini indices, and the variable with the lowest Gini index value was selected as the decision node. DTL divided the dataset by its branches and repeat the same process on every branch. A branch with zero Gini index becomes a leaf node, while a branch with Gini index larger than zero needs further splitting. DTL is run recursively in a similar way on the non-leaf branches, until all data were classified.

## Results

Electrocardiograms of patients with mitral stenosis (*n* = 155) were screened. In this cohort, 96 had atrial fibrillation on admission to our hospital without prior ECGs in sinus rhythm available for analysis. The remaining 59 patients were in sinus rhythm and were analyzed further. A graphical representation of the different *P*-wave indices is shown in Figure 1. The baseline characteristics of this cohort are shown in Supplementary Table 1. The median age was 59 [54–65] years old, and 13 patients (22%) were male.

A total of 27 patients developed new onset AF over a median follow-up of 58 [48–76] months. Patients with new onset AF had similar mean PWD (102 [95–118] vs. 101 [89–115] ms), minimum PWD (56 [40–68] vs. 52 [38–65]), maximum PWD (152 [132–164] vs. 136 [123–160] ms), *P*-wave dispersion (84 [62–116] vs. 82 [57–110] ms), standard deviation of PWD (28 [17–33] vs. 24 [16–33] ms), mean *P*-wave amplitude (0.11 [0.09–0.15] vs. 0.11 [0.09–0.13] mV), minimum *P*-wave amplitude (0.05 [0.03–0.07] vs. 0.05 [0.03–0.06] mV), maximum *P*-wave amplitude (0.18 [0.15–0.25] vs. 0.19 [0.16–0.23] mV), dispersion of *P*-wave amplitude (0.14 [0.10–0.17] vs. 0.14 [0.11–0.19] mV), standard deviation of *P*-wave amplitude (0.04 [0.03–0.06] vs. 0.04 [0.03–0.06] mV), mean *P*-wave area (0.12 [0.09–0.14] vs. 0.10 [0.09–0.13] Ashman units [40 ms × 0.1 mV]), minimum *P*-wave area (0.05 [0.04–0.07] vs. 0.05 [0.03–0.06] Ashman units), maximum *P*-wave area (0.22 [0.17–0.26] vs. 0.18 [0.14–0.26] Ashman units), dispersion of *P*-wave area (0.16 [0.11–0.20] vs. 0.12 [0.11–0.16] Ashman units), standard deviation of *P*-wave area (0.05 [0.04–0.06] vs. 0.04 [0.03–0.04] Ashman units), Neither *P*-wave initial force in V1 (PIFV1: 7.6 [3.7–11.7] vs. 3.6 [1.7–11.0] ms.mV) nor *P*-wave terminal force in V1 (PTFV1: 2.7 [0–6.9] vs. 3.8 [0–8.3] ms.mV) differed between the groups. Similarly, no difference in left atrial diameter was detected (4.9 [4.3–5.0] vs. 4.4 [3.9–4.9] cm, *P* = 0.1179). By contrast, *P*-wave area in V3 was significantly higher in the new onset AF group (1.0 [0.7–1.9] vs. 0.8[0.5–1.1]; *P* = 0.045).

The results of univariate logistic regression are shown in Supplementary Table 2. Age (odds ratio [OR]: 1.08 [1.01–1.16], *P* = 0.017), systolic blood pressure (OR: 1.03 [1.00–1.07]; *P* = 0.046), mean *P*-wave area in V3 (odds ratio: 3.97 [1.32–11.96], *P* = 0.014) were significant predictors of incident AF. Variables that achieved *P* < 0.10 in univariate logistic regression were included in the multivariable model (Supplementary Table 3). On multivariate analysis, age (OR: 1.08 [1.00–1.16], *P* = 0.037) and *P*-wave area in V3 (OR: 3.64 [1.10–12.00], *P* = 0.034) remained significant predictors of AF. Receiver-operating characteristic (ROC) analysis showed that the optimum cut-off for *P*-wave area in V3 was 1.45 Ashman units with an area under the curve of 0.65 for classifying new onset AF.

**Figure 3:**
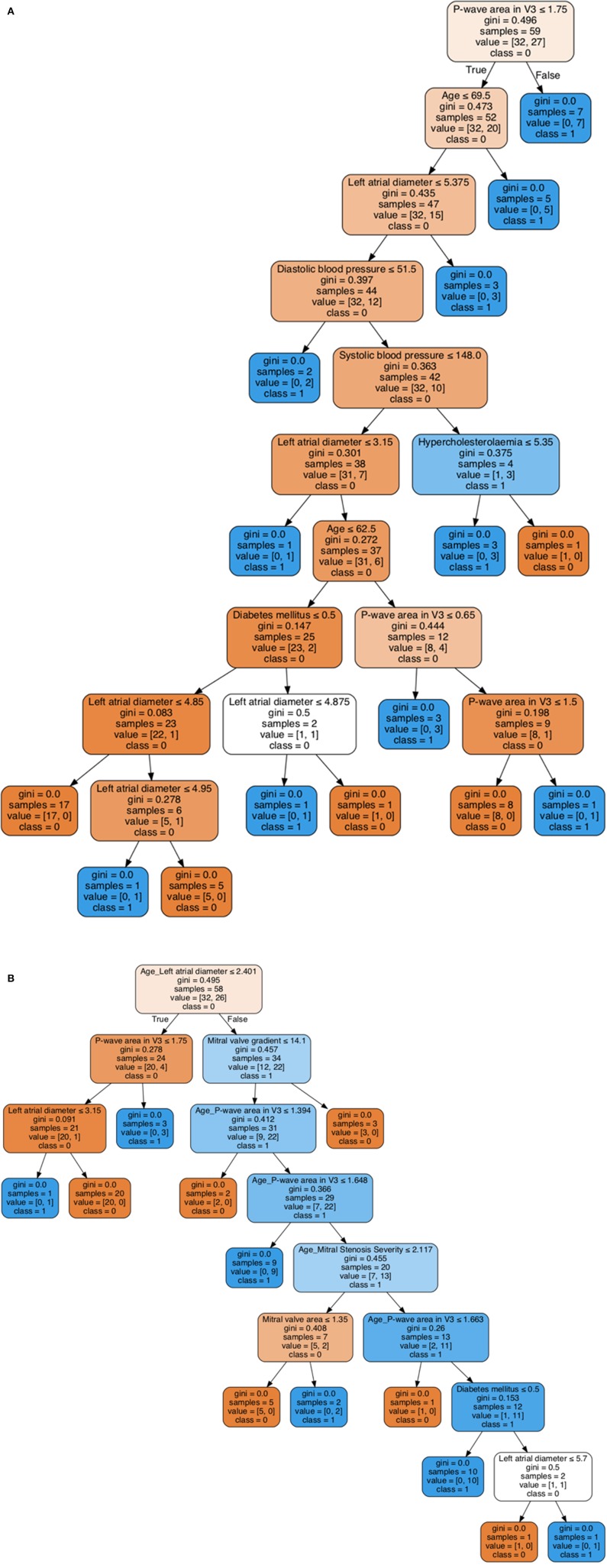
Visualization of decision tree learning with individual variables **(A)**. Visualization of decision tree learning with both individual and non-linear interaction variables **(B)**.

A decision tree learning (DTL) model was then employed to generate the decision rules based on only individual variables as shown in Figure 3A, while the decision rules generated by DTL model based on both individual and non-linear interaction items are shown in Figure 3B. In each model, 80% of the sample (*n* = 47) were randomly selected and the remaining 20% (*n* = 12) were used for validation. For the decision tree without interacting variables, *P*-wave area in V3 is the first predictor generated with a Gini index of 0.496 (Figure 3A). In addition, the interaction between age and left atrial diameter is the first variable in the generated decision rule with a Gini index of 0.495, while *P*-wave area in V3 as the second most important variable with Gini index 0.278 (Figure 3B). Both decision trees generated by machine learning can be used as an efficient tool for accurate risk stratification in mitral stenosis. The decision rule incorporating interactions between variables is more accurate.

To determine the out-of-sample prediction ability of the model, five-fold cross-validation was adopted. The evaluation metrics evaluated are accuracy, precision, recall, and F-measure. Comparisons of the prediction performance of logistic regression and DTL are shown in Supplementary Table 4. DTL with individual and non-linear variables outperforms the logistic regression for predicting incident AF in mitral stenosis. The non-linear variable formed by *P*-wave area in V3 and age was significantly predictive for classifying new onset AF outcome in mitral stenosis. We can also observe that several non-linear variables are more predictive than individual variables, indicating the importance of considering the non-linear patterns in clinical characteristics to improve the performance of predicting new onset AF.

## Discussion

The major findings of this study are that (i) a high proportion of patients with mitral stenosis had IAB, (ii) age and *P*-wave area in V3 predicted new onset AF, and (iii) a stepwise improvement in the predictive performance after incorporation of interaction variables and machine learning using a decision tree approach.

Prior studies have investigated alterations in *P*-wave morphologies and indices in non-valvular atrial fibrillation. Whilst some reports have dealt with the relationship between mitral stenosis and *P*-wave indices, few studies have examined whether these indices can predict new onset AF. Atrial electrophysiology in mitral stenosis is abnormal due to a complex process of electrophysiological remodeling. IAB is the conduction delay along the Bachmann's bundle between left and right atria, diagnosed by its characteristic ECG pattern (Agarwal et al., 2003; Bayes de Luna et al., 2012). Partial IAB and advanced IAB are defined as PWD ≥ 120 ms in the presence and absence of biphasic *P*-waves in the inferior leads. The association between IAB and supraventricular tachyarrhythmias, especially AF, is known as Bayés syndrome. Mitral stenosis is a major risk factor for AF through atrial dilatation with progressive structural remodeling and interstitial fibrosis, predisposing to re-entrant activity within the atrium (Markides and Schilling, 2003; O'neal et al., 2016). In addition to IAB, abnormal atrial electrophysiology can be detected by alterations in *P*-wave morphology on the electrocardiogram (ECG), including *P*-wave dispersion and abnormal *P*-wave terminal force in V1 (PTFV1) (Yamada et al., 1999; Dogan et al., 2004; Wong et al., 2004; Koide et al., 2008; Tsioufis et al., 2010; Yoshizawa et al., 2014). A previous study involving 30 mitral stenosis patients found that maximum *P*-wave duration and *P*-wave dispersion were significantly higher than patients without mitral stenosis (Guntekin et al., 2008). In the same previous study, baseline maximum and minimum PWDs and *P*-wave dispersion all correlated with mitral valve area and mean mitral gradient (Guntekin et al., 2008). Another study involving a prospective follow-up of 116 mitral stenosis patients similarly reported these associations, and additionally correlated these ECG parameters with increased pulmonary artery pressure, and a poor NYHA class (Yuce et al., 2011). Moreover, the extent to which atrial electrical abnormalities can predict incident AF remains less explored in these previous studies. Whilst previous studies have demonstrated the predictive value of various *P*-wave indices for incident AF or progression from paroxysmal to persistent AF (Koide et al., 2008), to date there are no studies specifically on their values in mitral stenosis. In our cohort, we utilized automated ECG measurements and found that *P*-wave area significantly predicted new onset AF.

In clinical practice, it is often useful to use cut-off values to identify categorize whether a patient is at high or low risk of adverse events. For example, a previous study identified that PWDs longer than 150 ms predicted AF recurrence after pulmonary isolation procedures (Jadidi et al., 2018). It should be noted that in the prior study, the ECG signals were amplified to 0.2–0.25 mV/cm before manual measurements were made, and the methodology therefore differs from that used here. In a cohort of patients with left atrial overload, area of the initial portion of the *P*-wave ≥ 65 μV.ms was associated with a four-fold increased risk of developing future AF (Ishida et al., 2010). In our study, the optimum cut-off for *P*-wave area in V3 was 1.45 Ashman units, with an area under the ROC curve of 0.65. Our novelty lies with the demonstrations that *P*-wave area can be used to predict incident AF in mitral stenosis and the use of machine learning approaches significantly improve outcome classification.

## Limitations

Some limitations should be recognized. Firstly, this was a single center study with a small sample size and retrospective analyses. Some inherent bias could affect the results. However, electronic health records in Hong Kong are comprehensive with accessible information across different hospitals within the public system and multiple follow-ups per year in the outpatient and inpatient settings. Secondly, the effects of drugs were not explored in the current study. Thirdly, left atrial diameter was the only available metric on atrial dimensions, as this was the only variable described in the echocardiography reports. Future work could explore (i) whether left atrial area, volume or volume index can predict incident AF and (ii) the potential effects on atrial reverse remodeling by drugs. Our main conclusion that *P*-wave area predicts incident AF needs to be validated by larger prospective studies.

## Conclusion

Atrial electrophysiological alterations in mitral stenosis can detected on the electrocardiogram. Age, systolic blood pressure, and mean PWD predicted new onset AF. A decision tree learning model significantly improve outcome prediction.

## Data Availability Statement

All datasets generated for this study are included in the article/Supplementary Material.

## Ethics Statement

The studies involving human participants were reviewed and approved by The Joint Chinese University of Hong Kong—New Territories East Cluster Clinical Research Ethics Committee. Written informed consent for participation was not required for this study in accordance with the national legislation and the institutional requirements.

## Author Contributions

GT: conception of study and literature search, figures, study design, data collection, data analysis, data contribution, manuscript drafting, and critical revision of manuscript. IL: literature search, data analysis, data contribution, manuscript drafting, and critical revision of manuscript. JZ and KLi: data analysis, manuscript drafting, and critical revision of manuscript. SL, YL, KLe, and TL: data interpretation, and critical revision of manuscript. AB: literature search, data analysis, critical revision of manuscript, and study supervision. QZ: literature search, figures, study design, data analysis, manuscript drafting, critical revision of manuscript, and study supervision.

## Conflict of Interest

The authors declare that the research was conducted in the absence of any commercial or financial relationships that could be construed as a potential conflict of interest.

## References

[B1] Agarwal Y. K. Aronow W. S. Levy J. A. Spodick D. H. (2003). Association of interatrial block with development of atrial fibrillation. Am. J. Cardiol. 91:882. 10.1016/S0002-9149(03)00027-4 12667579

[B2] Ariyarajah V. Puri P. Apiyasawat S. Spodick D. H. (2007). Interatrial block: a novel risk factor for embolic stroke? Ann. Noninvasive Electrocardiol. 12, 15–20. 10.1111/j.1542-474X.2007.00133.x 17286646 PMC6932538

[B3] Bayes de Luna A. Platonov P. Cosio F. G. Cygankiewicz I. Pastore C. Baranowski R.. (2012). Interatrial blocks. A separate entity from left atrial enlargement: a consensus report. J. Electrocardiol. 45, 445–451. 10.1016/j.jelectrocard.2012.06.029 22920783

[B4] Budeus M. Hennersdorf M. Perings C. Wieneke H. Erbel R. Sack S. (2005). Prediction of the recurrence of atrial fibrillation after successful cardioversion with *P* wave signal-averaged ECG. Ann. Noninvasive Electrocardiol. 10, 414–419. 10.1111/j.1542-474X.2005.00059.x 16255751 PMC6932341

[B5] Caldwell J. Koppikar S. Barake W. Redfearn D. Michael K. Simpson C.. (2014). Prolonged *P*-wave duration is associated with atrial fibrillation recurrence after successful pulmonary vein isolation for paroxysmal atrial fibrillation. J. Interv. Card. Electrophysiol. 39, 131–138. 10.1007/s10840-013-9851-1 24306110

[B6] Dogan A. Avsar A. Ozturk M. (2004). *P*-wave dispersion for predicting maintenance of sinus rhythm after cardioversion of atrial fibrillation. Am. J. Cardiol. 93, 368–371. 10.1016/j.amjcard.2003.09.064 14759395

[B7] Guntekin U. Gunes Y. Tuncer M. Gunes A. Sahin M. Simsek H. (2008). Long-term follow-up of P-wave duration and dispersion in patients with mitral stenosis. Pacing Clin. Electrophysiol. 31, 1620–1624. 10.1111/j.1540-8159.2008.01235.x 19067816

[B8] He J. Tse G. Korantzopoulos P. Letsas K. P. Ali-Hasan-Al-Saegh S. Kamel H.. (2017). *P*-wave indices and risk of ischemic stroke: a systematic review and meta-analysis. Stroke 48, 2066–2072. 10.1161/STROKEAHA.117.017293 28679858

[B9] Ishida K. Hayashi H. Miyamoto A. Sugimoto Y. Ito M. Murakami Y.. (2010). *P* wave and the development of atrial fibrillation. Heart Rhythm 7, 289–294. 10.1016/j.hrthm.2009.11.012 20133209

[B10] Jadidi A. Muller-Edenborn B. Chen J. Keyl C. Weber R. Allgeier J.. (2018). The duration of the amplified sinus-*P*-wave identifies presence of left atrial low voltage substrate and predicts outcome after pulmonary vein isolation in patients with persistent atrial fibrillation. JACC Clin. Electrophysiol. 4, 531–543. 10.1016/j.jacep.2017.12.001 30067494

[B11] Koide Y. Yotsukura M. Ando H. Aoki S. Suzuki T. Sakata K.. (2008). Usefulness of P-wave dispersion in standard twelve-lead electrocardiography to predict transition from paroxysmal to persistent atrial fibrillation. Am. J. Cardiol. 102, 573–577. 10.1016/j.amjcard.2008.04.065 18721514

[B12] Lankveld T. Zeemering S. Scherr D. Kuklik P. Hoffmann B. A. Willems S.. (2016). Atrial fibrillation complexity parameters derived from surface ECGs predict procedural outcome and long-term follow-up of stepwise catheter ablation for atrial fibrillation. Circ. Arrhythm. Electrophysiol. 9:e003354. 10.1161/CIRCEP.115.003354 26823480

[B13] Magnani J. W. Gorodeski E. Z. Johnson V. M. Sullivan L. M. Hamburg N. M. Benjamin E. J.. (2011). P wave duration is associated with cardiovascular and all-cause mortality outcomes: the National health and nutrition examination survey. Heart Rhythm 8, 93–100. 10.1016/j.hrthm.2010.09.020 20868770 PMC3046401

[B14] Markides V. Schilling R. J. (2003). Atrial fibrillation: classification, pathophysiology, mechanisms and drug treatment. Heart 89, 939–943. 10.1136/heart.89.8.939 12860883 PMC1767799

[B15] Martin Garcia A. Jimenez-Candil J. Hernandez J. Martin Garcia A. Martin Herrero F. Martin Luengo C. (2012). P wave morphology and recurrence after cardioversion of lone atrial fibrillation. Rev. Esp. Cardiol. 65, 289–290. 10.1016/j.rec.2011.04.020 21803476

[B16] Morris J. J. Jr, Estes, E. H. Jr, Whalen, R. E. Thompson H. K. Jr, Mcintosh, H. D. (1964). P-wave analysis in valvular heart disease. Circulation 29, 242–252. 10.1161/01.CIR.29.2.242 14119389

[B17] O'neal W. T. Venkatesh S. Broughton S. T. Griffin W. F. Soliman E. Z. (2016). Biomarkers and the prediction of atrial fibrillation: state of the art. Vasc. Health Risk Manag. 12, 297–303. 10.2147/VHRM.S75537 27486329 PMC4957677

[B18] Rezaian G. R. Rezaian S. Liaghat L. Zare N. (2007). P-wave dispersion in patients with rheumatic mitral stenosis. Int. J. Angiol. 16, 20–23. 10.1055/s-0031-1278239 22477244 PMC2733005

[B19] Rutkowski L. Jaworski M. Pietruczuk L. Duda P. (2014). The CART decision tree for mining data streams. Inf. Sci. 266, 1–15. 10.1016/j.ins.2013.12.060

[B20] Tse G. Lai E. T. Yeo J. M. Yan B. P. (2016). Electrophysiological mechanisms of bayes syndrome: insights from clinical and mouse studies. Front. Physiol. 7:188. 10.3389/fphys.2016.00188 27303306 PMC4886053

[B21] Tsioufis C. Syrseloudis D. Hatziyianni A. Tzamou V. Andrikou I. Tolis P.. (2010). Relationships of CRP and P wave dispersion with atrial fibrillation in hypertensive subjects. Am. J. Hypertens. 23, 202–207. 10.1038/ajh.2009.231 19942863

[B22] Wong T. Davlouros Periklis A. Li W. Millington-Sanders C. Francis Darrel P. Gatzoulis Michael A. (2004). Mechano-electrical interaction late after fontan operation. Circulation 109, 2319–2325. 10.1161/01.CIR.0000129766.18065.DC 15136502

[B23] Yamada T. Fukunami M. Shimonagata T. Kumagai K. Sanada S. Ogita H.. (1999). Dispersion of signal-averaged P wave duration on precordial body surface in patients with paroxysmal atrial fibrillation. Eur. Heart J. 20, 211–220. 10.1053/euhj.1998.1281 10082154

[B24] Yoshizawa T. Niwano S. Niwano H. Igarashi T. Fujiishi T. Ishizue N.. (2014). Prediction of new onset atrial fibrillation through P wave analysis in 12 lead ECG. Int. Heart J. 55, 422–427. 10.1536/ihj.14-052 25098176

[B25] Yuce M. Davutoglu V. Akkoyun C. Kizilkan N. Ercan S. Akcay M.. (2011). Interatrial block and P-terminal force: a reflection of mitral stenosis severity on electrocardiography. J. Heart Valve Dis. 20, 619–623. 22655490

