# Supplementary material for: P-Wave Area Predicts New Onset Atrial Fibrillation in Mitral Stenosis: A Machine Learning Approach
Source: Front Bioeng Biotechnol. 2020 May 15;8:479. doi: 10.3389/fbioe.2020.00479 (PMC7243705; doi:10.3389/fbioe.2020.00479)
Supplement: Supplementary file 1 [file Table_1.DOC]

**Supplementary Tables**

**Supplementary Table 1. Baseline characteristics of mitral stenosis patients included in this study.**

*Data are presented as median (interquartile range) or number (percentage) as appropriate. * Ashman units are given by 40 ms x 0.1 mV.*

| Variables | Value at baseline |
| --- | --- |
| Age | 66 (59-74) |
| Sex (male) | 36 (24) |
| Systolic blood pressure (mmHg) | 117 (108-133) |
| Diastolic blood pressure (mmHg) | 69 (63-75) |
| Diabetes mellitus | 4 (7) |
| Cholesterol | 4.7 (4.1-5.2) |
| Ischaemic heart disease | 10 (17) |
| Mitral valve area (cm^2^) | 1.1 (0.9-1.3) |
| Mitral valve gradient (mmHg) | 8 (6-12) |
| Mean P-wave duration (ms) | 102 (93-116) |
| Minimum P-wave duration (ms) | 52 (40-68) |
| Maximum P-wave duration (ms) | 139 (127-160) |
| P-wave dispersion (ms) | 84 (60-114) |
| Standard deviation of P-wave duration (ms) | 27 (17-33) |
| Mean P-wave amplitude (mV) | 0.11 (0.09-0.13) |
| Minimum P-wave amplitude (mV) | 0.05 (0.03-0.06) |
| Maximum P-wave amplitude (mV) | 0.19 (0.15-0.24) |
| Dispersion of P-wave amplitude (mV) | 0.14 (0.11-0.18) |
| Standard deviation of P-wave amplitude (mV) | 0.04 (0.03-0.06) |
| Mean P-wave area (Ashman units *) | 1.3 (1.0-1.6) |
| Minimum P-wave area (Ashman units) | 0.4 (0.2-0.6) |
| Maximum P-wave area (Ashman units) | 2.6 (2.0-3.3) |
| Dispersion of P-wave area (Ashman units) | 2.2 (1.6-3.0) |
| Standard deviation of P-wave area (Ashman units) | 0.7 (0.5-0.9) |
| P-wave terminal force in V1 (ms.mV) | 3.5 (0-8.2) |

**Supplementary Table 2**. Univariable logistic regression results

| Variable | Odds ratio | Std. Error | z value | Pr(>|z|) |
| --- | --- | --- | --- | --- |
| Sex (male) | 2.27 (0.64-8.03) | 1.464 | 1.28 | 0.202 |
| Age | 1.08 (1.01-1.16) | 0.036 | 2.38 | **0.017** |
| Systolic blood pressure | 1.03 (1.00-1.07) | 0.018 | 1.99 | **0.046** |
| Diastolic blood pressure | 1.03 (0.98-1.07) | 0.024 | 1.04 | 0.299 |
| Diabetes mellitus | 1.20 (0.16-9.14) | 1.243 | 0.18 | 0.860 |
| Hypercholesterolaemia | 1.16 (0.59-2.26) | 0.396 | 0.42 | 0.673 |
| Ischaemic heart disease | 2.95 (0.78-11.19) | 2.006 | 1.59 | 0.112 |
| Mitral valve area | 1.48 (0.28-7.95) | 1.270 | 0.46 | 0.646 |
| Mitral valve gradient | 0.96 (0.83-1.10) | 0.068 | -0.65 | 0.517 |
| P-wave frontal axis | 1.00 (0.98-1.02) | 0.010 | -0.05 | 0.963 |
| P-wave horizontal axis | 1.01 (1.00-1.02) | 0.006 | 1.13 | 0.257 |
| P-wave duration in V1 (positive component) | 1.00 (0.99-1.02) | 0.007 | 0.51 | 0.613 |
| P-wave amplitude in V1 (positive component) | 35.47 (0.49-2568.23) | 77.51 | 1.63 | 0.102 |
| P-wave area in V1 (positive component) | 1.34 (0.89-2.02) | 0.281 | 1.40 | 0.160 |
| P-wave duration in V1 (negative component) | 1.00 (0.98-1.01) | 0.007 | -0.19 | 0.851 |
| P-wave amplitude in V1 (negative component) | 1.80 (0.001-5586.95) | 7.395 | 0.14 | 0.886 |
| P-wave area in V1 (negative component) | 1.19 (0.55-2.57) | 0.468 | 0.43 | 0.664 |
| P-wave terminal force in V1 | 0.97 (0.87-1.08) | 0.053 | -0.56 | 0.577 |
| P-wave duration in aVR (positive component) | 0.98 (0.96-0.99) | 0.009 | -2.59 | **0.010** |
| P-wave amplitude in aVR (positive component) | 6322437 (126.84-3.15e+11) | 3.49e+07 | 2.84 | **0.005** |
| P-wave area in aVR (positive component) | 4.79 (1.85-12.38) | 2.321 | 3.23 | **0.001** |
| P-wave duration in aVR (negative component) | - (predicted perfectly) | - | - | - |
| P-wave amplitude in aVR (negative component) | 2.15e-06 (1.51e-36- 3.06e+24) | 0.00001 | -0.37 | 0.713 |
| P-wave area in aVR (negative component) | 0.10 (9.26e-06- 1180.91) | 0.498 | -0.47 | 0.635 |
| P-wave duration in aVR (total) | 1.00 (0.99-1.02) | 0.008 | 0.43 | 0.671 |
| P-wave amplitude in aVR (total) | 0.28 (0.00003-2506.17) | 1.294 | -0.28 | 0.783 |
| P-wave area in aVR (total) | 0.64 (0.36-1.15) | 0.191 | -1.50 | 0.134 |
| P-wave terminal force in aVR | 1 (predicted perfectly) | - | - | - |
| P-wave duration in V3 (positive component) | 0.98 (0.97-1.00) | 0.008 | -2.15 | **0.032** |
| P-wave amplitude in V3 (positive component) | 3.01e-06 (2.37e-11-0.383) | 0.00002 | -2.12 | **0.034** |
| P-wave area in V3 (positive component) | 0.19 (0.06-0.60) | 0.112 | -2.82 | **0.005** |
| P-wave duration in V3 (negative component) | 1.02 (1.00-1.04) | 0.0116 | 1.65 | 0.098 |
| P-wave amplitude in V3 (negative component) | 6.14e-08 (4.73e-17-79.80) | 6.57e-07 | -1.55 | 0.121 |
| P-wave area in V3 (negative component) | 0.128 (0.01-2.45) | 0.193 | -1.36 | 0.173 |
| P-wave duration in V3 | 1.00 (0.99-1.02) | 0.008 | 0.39 | 0.696 |
| P-wave amplitude in V3 | 17919.69 (0.07-4.74e+09) | 114150.1 | 1.54 | 0.124 |
| P-wave area in V3 | 3.97 (1.32-11.96) | 2.233 | 2.45 | **0.014** |
| P-wave terminal force in V3 | 1.31 (0.90-1.93) | 0.256 | 1.40 | 0.162 |
| Mean P-wave duration (positive component) | 1.00 (0.97-1.03) | 0.160 | 0.00 | 0.999 |
| Minimum P-wave duration (positive component) | 1.03 (0.99-1.07) | 0.020 | 1.38 | 0.168 |
| Maximum P-wave duration (positive component) | 1.00 (0.98-1.02) | 0.009 | 0.07 | 0.941 |
| P-wave dispersion (positive component) | 0.99 (0.98-1.01) | 0.009 | -0.59 | 0.553 |
| Standard deviation of P-wave duration (positive component) | 0.99 (0.94-1.05) | 0.027 | -0.25 | 0.801 |
| Mean P-wave duration (negative component) | 1.03 (0.98-1.08) | 0.026 | 1.16 | 0.248 |
| Minimum P-wave duration (negative component) | - (collinearity) | - | - | - |
| Maximum P-wave duration (negative component) | 1.00 (0.98-1.02) | 0.009 | 0.16 | 0.870 |
| P-wave dispersion (negative component) | 1.00 (0.98-1.02) | 0.009 | 0.16 | 0.870 |
| Standard deviation of P-wave duration (negative component) | 1.02 (0.97-1.07) | 0.251 | 0.88 | 0.378 |
| Mean P-wave amplitude (positive component) | 2283.68 (0.003- 1.71e+09) | 15763.5 | 1.12 | 0.263 |
| Minimum P-wave amplitude (positive component) | 4248667 (6.14e-08 - 2.94e+20) | 6.91e+07 | 0.94 | 0.348 |
| Maximum P-wave amplitude (positive component) | 5.81 (0.05-637.49) | 13.93 | 0.73 | 0.463 |
| Dispersion of P-wave amplitude (positive component) | 0.96 (0.77-1.20) | 0.109 | -0.38 | 0.704 |
| Standard deviation of P-wave amplitude (positive component) | 0.80 (0.32-2.00) | 0.374 | -0.47 | 0.638 |
| Mean P-wave amplitude (negative component) | 9.04e-14 (2.30e-34 - 3.55e+07) | 2.19e-12 | -1.24 | 0.214 |
| Minimum P-wave amplitude (negative component) | 0.15 (0.0002-83.75) | .479 | -0.59 | 0.555 |
| Maximum P-wave amplitude (negative component) | 98.61 (.0003- 3.84e+07) | 647.65 | 0.70 | 0.485 |
| Dispersion of P-wave amplitude (negative component) | 5.08 (0.04-597.05) | 12.36 | 0.67 | 0.504 |
| Standard deviation of P-wave amplitude (negative component) | 4651.88 (.00002- 1.10e+12) | 45754.98 | 0.86 | 0.391 |
| Mean P-wave area (positive component) | 1.29 (0.53-3.10) | 0.576 | 0.56 | 0.576 |
| Minimum P-wave area (positive component) | 4.11 (0.10-165.03) | 7.74 | 0.75 | 0.453 |
| Maximum P-wave area (positive component) | 1.01 (0.67-1.52) | 0.212 | 0.04 | 0.970 |
| Dispersion of P-wave area (positive component) | 1.03 (0.92-1.30) | 0.120 | 0.29 | 0.773 |
| Standard deviation of P-wave area (positive component) | 1.25 (0.56-2.79) | 0.512 | 0.54 | 0.589 |
| Mean P-wave area (negative component) | 0.10 (0.001-7.47) | 0.215 | -1.05 | 0.292 |
| Minimum P-wave area (negative component) | 0.84 (0.50-1.43) | 0.227 | -0.63 | 0.525 |
| Maximum P-wave area (negative component) | 3.28 (0.36-30.00) | 3.706 | 1.05 | 0.292 |
| Dispersion of P-wave area (negative component) | 1.19 (0.75-1.89) | 0.279 | 0.75 | 0.452 |
| Standard deviation of P-wave area (negative component) | 2.08 (0.36-12.10) | 1.868 | 0.81 | 0.416 |
| Mean P-wave duration (total) | 1.01 (0.98-1.04) | 0.015 | 0.75 | 0.454 |
| Minimum P-wave duration (total) | 1.01 (0.98-1.03) | 0.011 | 0.56 | 0.575 |
| Maximum P-wave duration (total) | 1.01 (0.99-1.02) | 0.009 | 0.57 | 0.567 |
| P-wave dispersion (total) | 1.00 (0.99-1.02) | 0.008 | 0.11 | 0.913 |
| Standard deviation of P-wave duration (total) | 1.00 (0.95-1.06) | 0.026 | 0.10 | 0.917 |
| Mean P-wave amplitude (total) | 2171.26 (.008 - 5.91e+08) | 13862.68 | 1.20 | 0.229 |
| Minimum P-wave amplitude (total) | 35360.72 (4.55e-06 - 2.75e+14) | 410888.9 | 0.90 | 0.367 |
| Maximum P-wave amplitude (total) | 8.59 (0.08 - 908.09) | 20.43 | 0.90 | 0.366 |
| Dispersion of P-wave amplitude (total) | 5.66 (0.10-325.85) | 11.71 | 0.84 | 0.402 |
| Standard deviation of P-wave amplitude (total) | 374.75 (0-4.14e+08) | 2660.85 | 0.83 | 0.404 |
| Mean P-wave area (total) | 0.64 (0.27-1.51) | 0.281 | -1.01 | 0.312 |
| Minimum P-wave area (total) | 3.69 (0.41-33.40) | 4.144 | 1.16 | 0.246 |
| Maximum P-wave area (total) | 0.82 (0.56-1.21) | 0.160 | -0.98 | 0.325 |
| Dispersion of P-wave area (total) | 0.78 (0.51-1.21) | 0.172 | -1.10 | 0.271 |
| Standard deviation of P-wave area (total) | 0.47 (0.12-1.90) | 0.334 | -1.06 | 0.288 |
| Mean P-wave terminal force | 1.51 (0.83-2.78) | 0.470 | 1.34 | 0.179 |
| Minimum P-wave terminal force | - (collinearity) | - | - | - |
| Maximum P-wave terminal force | 1.02 (0.93-1.11) | 0.047 | 0.33 | 0.739 |
| Dispersion of P-wave terminal force | 1.02 (0.93-1.11) | 0.047 | 0.33 | 0.739 |
| Standard deviation of P-wave terminal force | 1.08 (0.81-1.43) | 0.155 | 0.53 | 0.598 |
| Left atrial diameter | 1.89 (0.82-4.36) | 0.807 | 1.50 | 0.135 |
| *, ** and *** were used to denote P < 0.05, 0.01 and 0.001, respectively. | | | | |

**Supplementary Table 3**. Multivariable logistic regression results

| Variable | Odds ratio | Std. Error | z value | Pr(>|z|) |
| --- | --- | --- | --- | --- |
| Age | 1.08 (1.00-1.16) | 0.039 | 2.08 | **0.037 *** |
| Systolic blood pressure | 1.01 (0.98-1.05) | 0.019 | 0.74 | 0.457 |
| P-wave area in V3 | 3.64 (1.10-12.00) | 2.215 | 2.12 | **0.034 *** |
| *, ** and *** were used to denote P < 0.05, 0.01 and 0.001, respectively. Adjusted for age, systolic blood pressure, P-wave area in V3. | | | | |

**Supplementary Table 4**. Model performance to predict new onset AF in mitral stenosis

| Model | Accuracy | Precision | Recall | F-measure |
| --- | --- | --- | --- | --- |
| LR with individual variables | 0.7272 | 0.7096 | 0.7074 | 0.7085 |
| LR with both individual and interaction variables | 0.7934 | 0.7577 | 0.7935 | 0.7752 |
| DTL with individual variables | 0.8191 | 0.8182 | 0.7933 | 0.8056 |
| DTL with both individual and interaction variables | 0.8391 | 0.8413 | 0.8301 | 0.8357 |
